# Optimization of a Piezoelectric Energy Harvester and Design of a Charge Pump Converter for CMOS-MEMS Monolithic Integration †

**DOI:** 10.3390/s19081895

**Published:** 2019-04-21

**Authors:** Marcos Duque, Edgardo Leon-Salguero, Jordi Sacristán, Jaume Esteve, Gonzalo Murillo

**Affiliations:** 1 Department of Micro and Nanoengineering, Instituto de Microelectrónica de Barcelona IMB-CNM (CSIC), Campus UAB Bellaterra, 08193 Barcelona, Spain; Marcos.Duque@imb-cnm.csic.es (M.D.); Jordi.sacristan@imb-cnm.csic.es (J.S.); Jaume.Esteve@imb-cnm.csic.es (J.E.); 2 Postgrado en Nanotecnología, Universidad de Sonora (Unison), Hermosillo, Sonora 83000, Mexico; eleonsal@gmail.com

**Keywords:** IoT, MEMS, energy harvesting, monolithic integration, piezoelectric, CMOS, charge pump, self-powered, power management, AlN

## Abstract

The increasing interest in the Internet of Things (IoT) has led to the rapid development of low-power sensors and wireless networks. However, there are still several barriers that make a global deployment of the IoT difficult. One of these issues is the energy dependence, normally limited by the capacitance of the batteries. A promising solution to provide energy autonomy to the IoT nodes is to harvest residual energy from ambient sources, such as motion, vibrations, light, or heat. Mechanical energy can be converted into electrical energy by using piezoelectric transducers. The piezoelectric generators provide an alternating electrical signal that must be rectified and, therefore, needs a power management circuit to adapt the output to the operating voltage of the IoT devices. The bonding and packaging of the different components constitute a large part of the cost of the manufacturing process of microelectromechanical systems (MEMS) and integrated circuits. This could be reduced by using a monolithic integration of the generator together with the circuitry in a single chip. In this work, we report the optimization, fabrication, and characterization of a vibration-driven piezoelectric MEMS energy harvester, and the design and simulation of a charge-pump converter based on a standard complementary metal–oxide–semiconductor (CMOS) technology. Finally, we propose combining MEMS and CMOS technologies to obtain a fully integrated system that includes the piezoelectric generator device and the charge-pump converter circuit without the need of external components. This solution opens new doors to the development of low-cost autonomous smart dust devices.

## 1. Introduction

The Internet of Things (IoT) has become a trending topic in the last years [1,2,3,4]. This concept allows the interconnection of thousands of wireless sensors to acquire ambient information and take decisions without human interaction. Nevertheless, the energy dependence of the wireless sensor nodes, limited by the capacity of batteries, is one of the most important barriers to the global implementation of the IoT. A promising alternative is to recover energy from the environment, in the way of heat, light, or mechanical energy [5,6,7]. This concept allows these devices to be self-sufficient. Ambient vibrations can be converted into electrical energy by using piezoelectric [8,9,10,11,12,13], triboelectric [14,15,16], electromagnetic [17], and electrostatic transducers [18,19,20]. Among them, piezoelectric transducers are considered an attractive option due to their high energy density, ease of integration, and durability. Piezoelectric MEMS allow device miniaturization, piezoelectric actuators with low operation voltages, easy implementation of high frequency, and temperature-stable resonant devices, and the possibility of harvesting energy from mechanical sources [21,22,23,24].

A piezoelectric generator provides an alternating-current (AC) electrical signal that needs to be rectified to get a direct current (DC). A simple energy harvesting circuit consists of a diode-based rectifier (AC-DC) and a DC-DC converter, such as the LTC3588 IC from Linear Technologies. The addition of a DC-DC converter has been shown to improve the conversion efficiency by a factor of seven. A more specialized power management system for piezoelectric generators is the “synchronized switch harvesting on inductor” (SSHI) circuit [25,26,27]. It consists of a switching device in parallel with a piezoelectric element. The device is composed of a switch and an inductor connected in series, and it allows a higher efficiency for the rectification stage. The inductance is by far the largest component of the power management circuit. This is a critical limitation for the circuit miniaturization; in addition, inductances of such high values are relatively expensive. A charge pump circuit is an alternative to the SSHI that only uses capacitors [28,29]. It is a switching regulator that delivers the power by only charging and discharging capacitors. It is appropriate for an application that has low currents and a moderate input-to-output voltage difference. From the energetic point of view, it is not as efficient as an SSHI converter, but it allows a full monolithic integration.

In addition, device encapsulation and packaging usually represents a large part of the manufacturing costs of MEMS devices and integrated circuits [30,31,32]. For these reasons, our goal is to monolithically combine MEMS and CMOS technologies to obtain a fully integrated system comprising of a piezoelectric generator and its associated converter circuit [33,34,35,36,37].

## 2. Piezoelectric MEMS Energy Harvester

### 2.1. Device Specifications and Fabrication Process

There are different types of MEMS structures, such as cantilevers, clamped-clamped beams, or diaphragms. Among them, a cantilever generates a higher average strain for a given input force, which in turns results in a higher power output when used as a piezoelectric generator. Furthermore, a cantilever has the lowest resonance frequency for a given size [38]. For this reason, the cantilever structure was chosen for our piezoelectric transducer.

**Figure 1:**
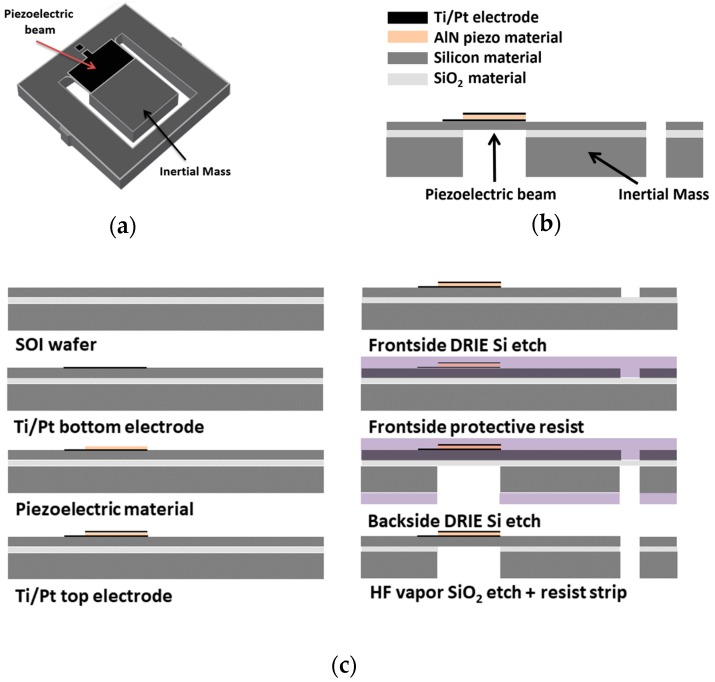
(**a**) Functional device configuration: A cantilever structure with a tip mass and a piezoelectric material deposited on top of the beam; (**b**) cross-section sketch of the cantilever structure; (**c**) different steps of the fabrication process.

The functional schematic of our piezoelectric MEMS energy harvester is shown in Figure 1a. Our energy harvesting devices are based on an silicon-on-insulator (SOI) silicon wafer (silicon: 15 µm—SiO_2_: 1 µm—silicon: 500 µm) [39,40]. The device layer is used to define the cantilever beam and the bulk layer is used for the inertial mass (Figure 1b). On top of the cantilever beam, a 1-µm-thick layer of aluminium nitride (AlN) forms the piezoelectric transducer. The piezoelectric layer is sandwiched between two bilayer metal electrodes of Ti/Pt. In most applications, the frequency of the ambient vibrations is lower than 1 KHz [41]. For this reason, we have used an inertial tip mass to decrease the structure’s natural frequency.

**Figure 2:**
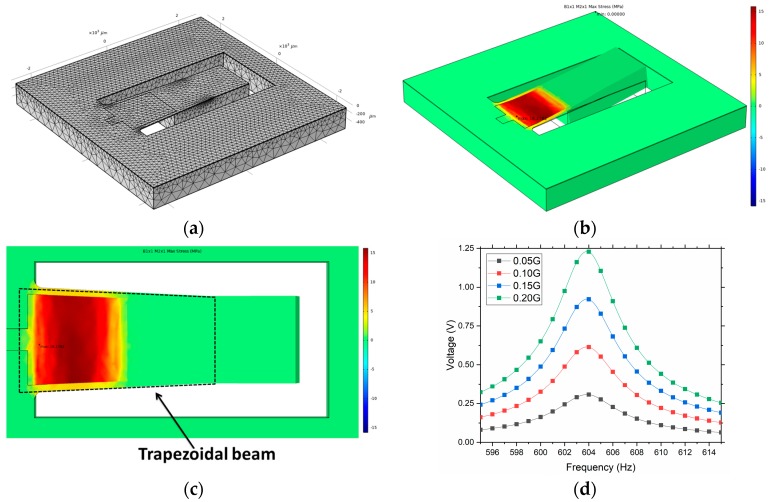
Simulated results of the piezoelectric device by using COMSOL multiphysics: (**a**) Mesh used in the simulation; (**b**) 3D FEM simulation of the stress distribution in the cantilever beam and (**c**) top view of the structure with the trapezoidal beam shape detailed (arbitrary units); (**d**) maximum open-circuit voltage that can be generated by the piezoelectric generator submitted to a frequency sweep, at different values of the input acceleration ranging from 0.05 G to 0.20 G.

The fabrication process of our devices is depicted in Figure 1c. The thin layer of Ti/Pt is deposited by radio-frequency reactive sputtering to define the two device electrodes. This same technique is used to deposit the AlN layer of 1 µm. The device structure is defined by several steps of photolithography and deep reactive-ion etching (DRIE). Finally, a wet etch based on hydrofluoric acid (HF) is used to remove part of the buried oxide layer and, consequently, to release the cantilever beam [35,40]. In this work, we focused on the PEH-B1X1M2X1 device, shown in Figure 2a–c, with a mass area of 1 mm × 2 mm and a cantilever area of 1 mm × 1 mm.

### 2.2. Design and FEM Simulations

The designed devices have been simulated with COMSOL Multiphysics to compare the expected results with the electrical characterization performed after manufacturing. The used modules for this simulation are the AC/DC module (electrostatics, electrical circuits) and the structural mechanics module (solid mechanics and piezoelectric devices). The materials selected are silicon (single-crystal, anisotropic) and aluminum nitride, with the default values provided by the software. Figure 2a shows the PEH-B1X1M2X1 device model with a swept mesh for the piezoelectric material and a tetrahedral mesh for the rest of structure. In this study, a fine mesh with 20,925 elements was utilized. The stress distribution in the structure, by using the Eigenfrequency study, was computed to achieve a uniform distribution over the entire surface of the piezoelectric layer (Figure 2b,c). In this way, a higher charge density is generated and therefore, a more efficient device can be obtained. After some careful design and simulation processes, beams with trapezoidal shapes and rounded corners were designed to achieve a homogeneous stress over the entire surface and avoid stress concentration in the corners (Figure 2c) [42]. Figure 2d shows the open-circuit voltage generated by the structure submitted to a frequency sweep of the excitation vibration at different values of acceleration. The open-circuit configuration is used to measure the maximum voltage that can be generated by the piezoelectric generator. Our simulation results show the resonant frequency of the device and the maximum open-circuit voltage that can be generated by the piezoelectric generator at different input accelerations.

Using the same structure, but without a trapezoidal shape and rounded corners, we obtained an open-circuit voltage of around 0.48 V for an acceleration of 0.1 G [39]. In our simulation, shown in Figure 2d, a voltage of 0.625 V was obtained with a homogeneous stress over the entire surface. Therefore, an increase of 30% in the voltage is expected by using this optimized design. 

### 2.3. Device Manufacturing

**Figure 3:**
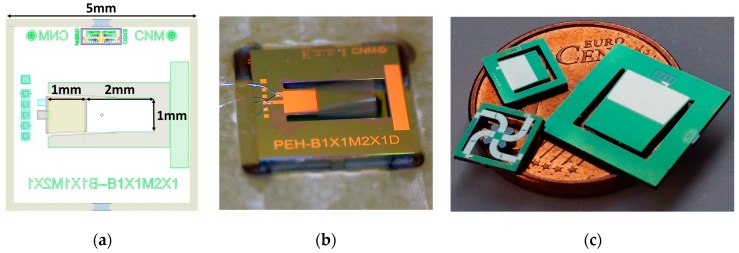
(**a**) Layout of a piezoelectric microgenerator (5 mm × 5 mm × 0.516 mm); (**b**) resonance motion of the fabricated device captured with a microscope camera; (**c**) different designs of manufactured piezoelectric microgenerators on top of a one cent coin.

**Figure 4:**
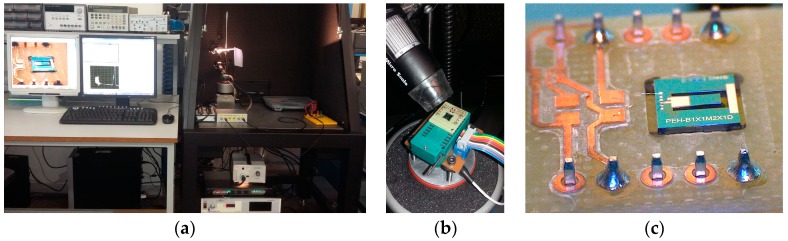
(**a**) Characterization setup for the piezoelectric MEMS devices; (**b**) microscope camera and DIP ZIF socket to anchor the DUT; (**c**) fabricated piezoelectric microgenerator bonded to a PCB and mounted on the shaker.

Several devices, shown in Figure 3c, with different geometries and designs were manufactured expecting a high power density. After manufacturing, every microgenerator was placed and bonded in a printed-circuit board (PCB) to perform the electrical characterizations (Figure 4c). The fabrication process for these devices is patented and licensed to ENERGIOT Devices SL [42,43,44], which is commercially exploiting this technology. The layout of the PEH-B1X1M2X1D device can be seen in Figure 3a. The resonance motion of this device was captured with a microscope camera (Figure 3b). Figure 3c shows some fabricated devices with different designs and sizes, demonstrating high miniaturization and customization capabilities.

### 2.4. Electromechanical Characterization

An electrodynamic shaker was used to emulate environmental vibrations at different input acceleration magnitudes (Vibration Testing Controller VR9500, Vibration Research, Jenison, MI, USA). This was controlled through a MATLAB program that allows automatizing, acquiring, and processing the voltage measurements at different frequencies and acceleration values (Figure 4a,b). All this data is measured by an acquisition system (National Instruments PCI-6132 and BNC-2110, National Instruments, Austin, TX, USA) with very high internal impedance to avoid charge leakages (100 MΩ). A special test PCB layout was designed to provide physical support and electrical connection to the chip (Figure 4c). The fabrication was carried out using a circuit board plotter. Finally, two wires were bonded to the PCB to obtain an electrical connection to the mounted device under test (DUT). A dual in-line package (DIP) zero insertion force (ZIF) socket was utilized to anchor the DUT to the shaker.

**Figure 5:**
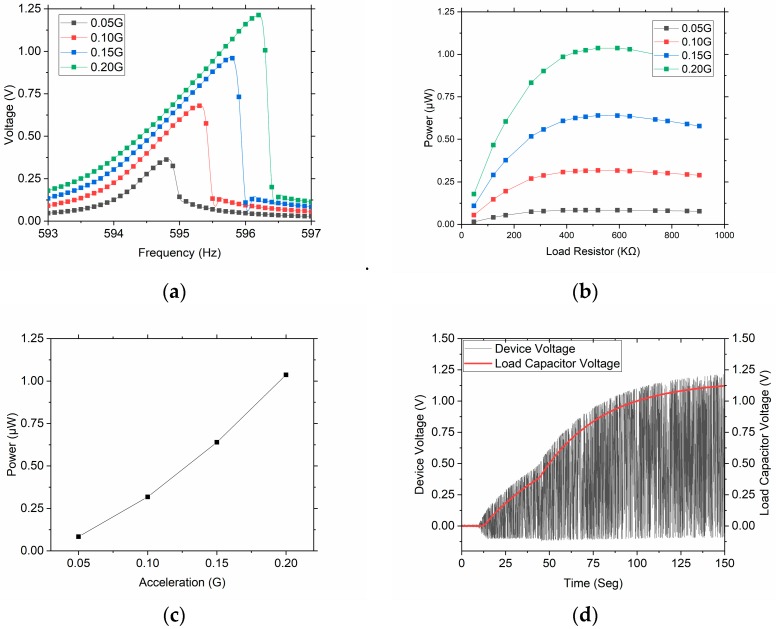
Graphs of the electrical characterization of the piezoelectric generator: (**a**) Maximum open-circuit voltage that can be generated by the piezoelectric generator submitted to a frequency sweep at different acceleration amplitudes; (**b**) dependence of the generated power with the value of the load resistor; (**c**) maximum power that can be generated at each of the accelerations; (**d**) voltage vs. time by using a Schottky diode bridge and a charge capacitor of 10 µF with an acceleration of 0.2 G, showing the charge collection and the energy increase in the capacitor.

Three different electrical characterizations were performed to demonstrate the correct operation of our piezoelectric MEMS generators (Figure 5). The open-circuit voltage of the piezoelectric generator was measured while performing a frequency sweep with accelerations ranging from 0.05 G to 0.2 G. This characterization allows the measurement of the maximum voltage that the piezoelectric device can generate at its resonance frequency (Figure 5a). The second characterization was performed by fixing a certain acceleration magnitude and sweeping the value of the load resistor. In this way, the value of the load resistor for an optimal power generation and the maximum generated power at each acceleration magnitude can be found (Figure 5b,c). Finally, a Schottky diode bridge and a load capacitor of 10 μF were connected to the piezoelectric generator subject to a constant acceleration of 0.2 G. The increase of the capacitor voltage by using this simple rectification circuit can be observed (Figure 5d). 

As shown in Figure 5a, a resonance peak ranging from 594.5 Hz to 596.5 Hz, depending on the acceleration magnitude, is clearly identifiable. This variation in the natural frequency is due to a non-linear response of the device, hypothetically caused by the very large amplitudes reached at higher accelerations. The quality factor, Q, is also affected by this non-linear effect, which also alters the structural damping [45,46,47]. The measured resonance frequencies are quite close to those simulated (604 Hz) by FEM and are shown in Figure 2d. Different load resistors, from 50 KΩ to 950 KΩ, were used to find the optimal value where the extracted power reaches a maximum value of 0.32 μW and 1.03 μW for a 0.1 G and 0.2 G of acceleration, respectively (Figure 5b,c). 

Regarding our previous work that used a similar structure, but without trapezoidal shapes or rounded corners [39], an increase in the power of 60% was obtained. The volume of this device was 12.9 mm^3^ (5 mm × 5 mm × 0.516 mm) for the complete chip, and 1.54 mm^3^ (1 mm × 3 mm × 0.516 mm) for the functional structure composed by the cantilever and the inertial mass (Figure 3a). Therefore, the effective power density for this energy harvester, using the data shown in Figure 5, is 665.4 µW/cm^3^ for a vibration acceleration of 0.2 G and a frequency of ~595 Hz. In previous works [48,49], the volume for the functional structures were 32.2 mm^3^ (8.2 mm × 7 mm × 0.535 mm) and 18.85 mm^3^ (6 mm × 7.8 mm × 0.402 mm), respectively. Their maximum power values generated were 3.0 µW and 29.7 µW for accelerations of 0.2 G and 0.5 G, respectively. Thence, the corresponding effective power densities were 93.1 µW/cm^3^ and 1575.5 µW/cm^3^. Our devices offer potential for a larger power density. Alternatively, they perform better in terms of the power density per unit of excitation amplitude.

## 3. Converter Circuit

The purpose of this circuit is the conversion of the alternating current (AC) signal generated by the piezoelectric MEMS and the adaptation of the output voltage to power a utility (i.e., wireless sensor node for IoT). In our case, contrary to typical macroscopic piezoelectric generators, the AC voltage generated is smaller than the one required by the final application. In addition, as the key goal, this circuit must be fully able to be integrated. For these reasons, our proposed circuit is a charge pump, suitable to be implemented in a monolithic system comprised by the converter circuit for energy collection and the piezoelectric resonant MEMS generator.

**Figure 6:**
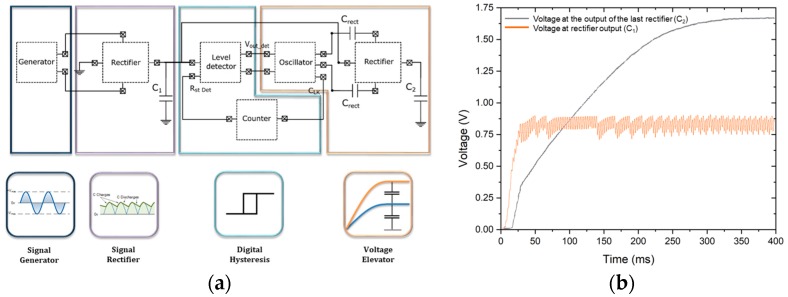
(**a**) Schematic of the converter circuit, showing the different function blocks; (**b**) graph of the simulated open-circuit voltages at capacitor, C_1_ and C_2_, for the piezoelectric MEMS generator connected to the converter circuit.

The converter circuit, shown in Figure 6a, consists of several functional blocks. The generator block is a model of the equivalent circuit of our piezoelectric. The function of the rectifier block is the conversion of the AC current to the direct current (DC) in order to charge the first capacitor (C_1_). The level detector waits for a certain threshold voltage in the first capacitor to power the oscillator once reached. Its function is to avoid energy consumption while charging the first storage capacitor. Then, the oscillator produces a periodic square wave of 5 kHz, by converting the DC voltage from the first capacitor [50]. The outputs of the oscillator are connected to another rectifier through two capacitors, in order to double the voltage in the second capacitor. The counter block is utilized as a timer to let the capacitor, C_1_, to be charged again [51]. Its function is to reset the level detector every *n* cycles of the oscillator.

The most energy consuming part of the converter circuit is the oscillator that forms the voltage elevator stage, shown in Figure 6a. The entire converter circuit consumes 1.875 µA from which the oscillator stage uses 825 nA. This corresponds to 44% of the total consumption. The instantaneous power generated by the piezoelectric generator is lower than the consumption of the oscillator. For this reason, we need a first block that rectifies and collects charge in a first capacitor (C_1_) and additional voltage elevator blocks that allow the increase of the final voltage (Figure 6b). Once the energy accumulated is sufficiently large, the remaining components of the charge pump are powered. For this to be achieved, we integrated a level detector and a counter. This working principle is compatible with the typical operation of the wireless sensor nodes used for IoT, where the duty cycle is very low (i.e., the device is normally in stand-by mode and only operates periodically for short cycles of time). 

The designs and simulations of the converter were performed by using CADENCE-Virtuoso 6.1.6-64b. We selected Silterra C18G—180 nm CMOS logic 1.8 V/3.3 V as the technology for this project. This is especially interesting because they can integrate AlN as piezoelectric material in its standard CMOS processes. Furthermore, Silterra technology offers an optimal combination of performance in terms of power, speed, and gate density. 

**Figure 7:**
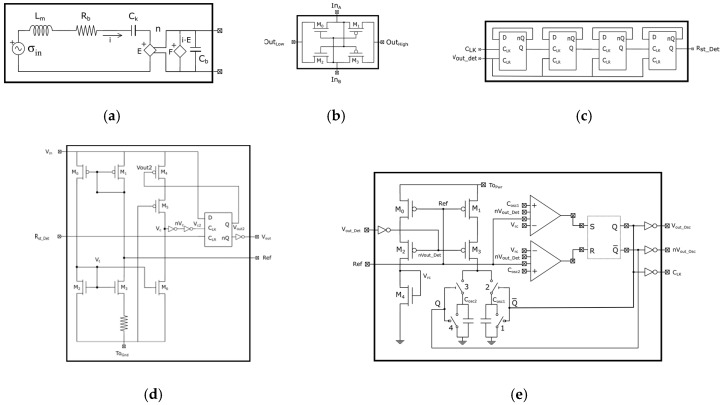
Schematic of the different blocks that constituent the converter circuit: (**a**) Generator circuit; (**b**) rectifier; (**c**) counter; (**d**) level detector; (**e**) oscillator.

The model of the equivalent piezoelectric circuit developed by S. Roundy [23] consists in modelling both the mechanical and electrical behaviour of the piezoelectric system as circuit elements. This block solves the simplified differential motion equation (i.e., the lumped parameter model of the forced damped spring-mass system with piezoelectric transduction) and generates an AC signal from a mechanical motion. The use of an ideal model containing only an AC source, which provides the corresponding open-circuit voltage at the resonance frequency, is a common alternative to model these energy harvesters. However, due to its simplicity, the simulation results are not realistic and the design of the converter circuit might not work properly. An ideal transformer is used by S. Roundy to model and represent the piezoelectric coupling. In this case, as shown in Figure 7a, this transformer was replaced by two dependent sources, thus avoiding modelling issues in different electrical simulation software, where the ratio, *n*, of the transformer depends on the inverse of the Young’s modulus, C_k_, and the capacitance of the piezoelectric layer, C_b_. These sources, E and F, represent the effects produced by the voltage on the mechanical response of the device and the generated current as a result of the mechanical stress, respectively. The voltage source, σ_in_, models the mechanical stress produced by the ambient vibration. The inductive element, L_m_, represents the inertial mass of the generator, and relates stress to the second derivate of strain. Finally, R_b_ symbolizes the mechanical damping of the system.

For the rectifier circuit, shown in Figure 7b, as input, In_A_, is higher than In_B_, the transistors, M_1_ and M_2_, are on and the input, In_A_, is connected to the output, Out_High_, and In_B_ is connected to Out_Low_. In contrast, as the input, In_B_, is higher than In_A_, the transistors, M_0_ and M_3_, are on and In_A_ and In_B_ are connected to Out_Low_ and Out_High_, respectively. Therefore, a full-wave rectified sinusoidal signal is generated.

To prevent the oscillator from working all the time and consuming power, a counter formed by D flip-flops was implemented (Figure 7c). This counter receives the input from the oscillator output signal and once it counts 16 level changes at the input of the first flip-flop, the last flip-flop changes its level. The output of this counter (R_st_det_) is connected to the reset input of the level detector (R_st_det_). In this way, every 16 cycles of the oscillator, the detector will be reset.

Figure 7d shows the schematics of the level detector circuit. The transistors, M_0_, M_1_, M_2_, and M_3_, form a current source from which the reference for the oscillator is taken. The D flip-flop always has its data input, D, connected to V_in_ (i.e., the power supply corresponding to the voltage of the first capacitor, C_1_). In this way, D is always set to a logic “1”. From the beginning, the D flip-flop starts with its outputs (Q, nQ) = (0,1), therefore, V_out2_ = ”0” and the M_4_ transistor enables the operation of the current comparator, implemented with M_3_ and M_5_. The C_LK_ input of the flip-flop is connected to V_c2,_ so the flip-flop changes its output state and V_out2_ = ”1”. Hence, at the output of the detector, the voltage of the input, V_in_, is obtained, and the M_4_ transistor goes off. The fact that the M_4_ transistor is off implies that the detector stops consuming power. Once the detector is activated and the output is connected to V_in_ (as shown in Figure 6a), the only way to change its state is to restart it through the C_LR_ input. The C_LR_ input resets the D flip-flop and causes the detector output to be set to “0”and M_4_ goes on again to start a new detection cycle. 

The oscillator circuit is shown in Figure 7e, where the schematic of the circuit that generates V_rc_ can be seen on the left side. This signal is used as a reference for the comparator to detect when the capacitor connected to the input of each comparator is charged. Once nV_out_det_ becomes ”0”, M_2_ starts driving and the voltage reference, V_rc_, is obtained at the drain of M_4_. With this configuration, we can obtain a voltage reference and the transistors, M_0_, M_1_, M_2_, M_3_, and M_4_, that form a current source do not consume power while the oscillator is not working. On the right side, we can see the schematic of the circuit that performs the charge and discharge of the capacitors. Four switches are formed by two PMOS transistors (switches 2 and 3) and two NMOS transistors (switches 1 and 4). It is assumed that at the initial operation state, the outputs of the SR flip-flop are (Q, nQ) = (0, 1). With this combination of outputs, switches 1 and 3 are closed, causing the capacitor, C_ap2_, to charge and the capacitor, C_ap1_, to discharge. When the capacitor, C_ap2_, reaches the reference value (V_rc_), the output of the second comparator changes its state. Likewise, when the capacitor, C_ap1_, is discharged and decreases below V_rc_, the output of the first comparator change its state. This forces the outputs to be (Q, nQ) = (1, 0) and switches 1 and 3 to open and switches 2 and 4 to closed, the originating capacitor, C_ap1_, to charge and the capacitor, C_ap2_, to discharge. When the capacitor, C_ap1_, reaches the reference value, V_rc_, the output of the first comparator changes its state. In this way, the outputs of the comparators will oscillate from one state to another, generating a square wave signal. The inverters connected between the outputs of the flip-flop and the output of the oscillator (nV_out_osc_, V_out_osc_, and C_LK_) avoid the current flow towards to the output of the oscillator and therefore, eliminates the associated power consumption.

**Figure 8:**
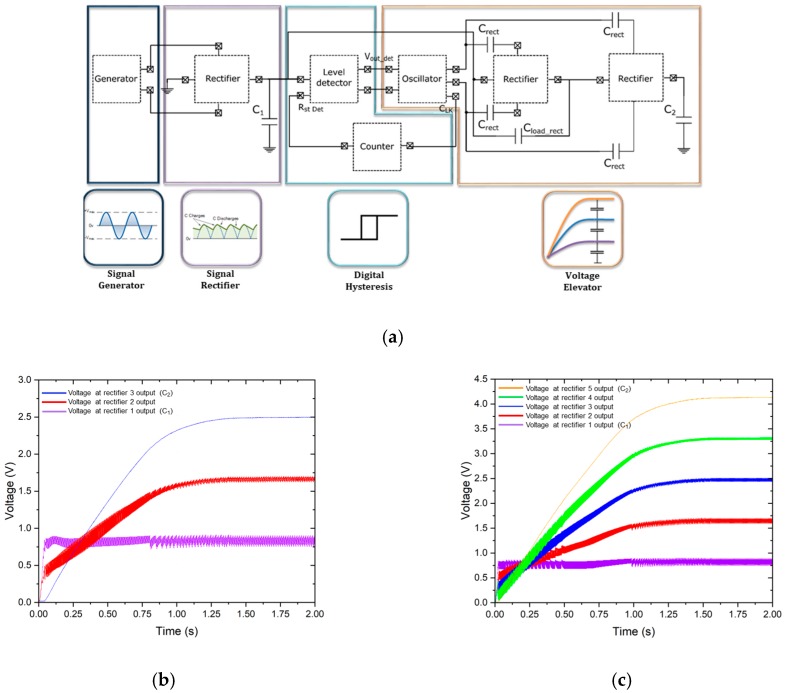
(**a**) Schematic of the converter circuit with *n* rectifier stages; (**b**,**c**) simulated output voltages with a converter circuit with (**b**) three and (**c**) five stages and a value of 20 nF for the capacitors, C_1_ and C_2_, when connected to the piezoelectric generator and submitted to an ambient vibration acceleration of 0.1 G.

In order to increase the output voltage to the desired value, additional rectifiers and the associated capacitors can be added at the voltage elevator stage. Figure 8 shows the simulated voltages at the output (V_C2_) for different converter circuits with three and five stages. Each time a rectifier is added, the time to reach the desired output voltage is doubled. This is because each time we transfer the energy from one capacitor to another, half of the energy is lost in that transfer. This is a rather inefficient strategy, but it is principally the only way of allowing a full monolithic integration.

## 4. Application Example

The proposed energy harvesting has potential applications related to the use of self-powered sensor networks for smart environments and predictive maintenance. One specific case is connected to the railway sector, in order to extend the maintenance intervals and to allow predictive maintenance of wheel bearings, gearboxes, and motors. SKF, a leading global technology provider and partner of the EnSO project (Energy for Smart Objects, Contract No. 692482), has provided us with different measurements of vibrations present in trains in operation. The vibration spectra of these measurements show accelerations from 0.05 G to 0.75 G with frequencies ranging from 100 Hz to 1000 Hz. Our devices can be designed to work in this range of frequencies and therefore, they are particularly suitable for this application.

**Figure 9:**
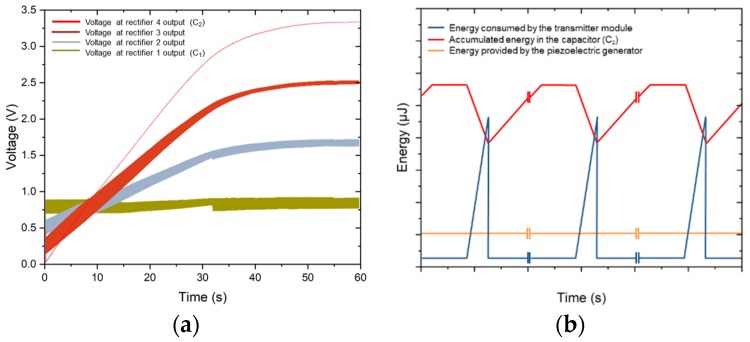
(**a**) Simulated output voltages with a converter circuit with four rectifying stages, 20 nF for capacitor (C_1_), and 1 μF for capacitor C_2_; (**b**) typical duty cycle of a wireless sensor node powered by energy harvesting.

In order to simulate the voltages and currents in the internal components of the converter during the long charge periods, a long transient computation with a very fine resolution is needed. For this reason, the previous simulations were performed with a value of 20 nF for the two capacitors (C_1_ and C_2_) and an acceleration of 0.15 G. However, this C_2_ capacitance value is not enough to power a wireless node sensor. A new simulation of the converter was carried out with four rectifying stages and capacitance values of 20 nF and 1 μF for C_1_ and C_2_, respectively (Figure 9a). This single simulation required a high capacity of calculation (159 GB of data) and an intensive and continuous use of computational resources (17 h of simulation with a dedicated server of 10 cores and 128 GB of RAM).

It can be seen that once the capacitance value of the final capacitor (C_2_) is increased to 1 μF, the final voltage does not change, demonstrating that the converter works correctly regardless of the capacitance value of the load capacitor. The energy accumulated in C_2_ at the end of the simulation for a capacitor of 1 μF and a potential difference of 3.325 V is 5.53 μJ.

A typical duty cycle of a wireless sensor node powered by energy harvesting is shown in Figure 9b. Hypothetically, the input energy flow generated by a piezoelectric harvester is continuous. The harvested energy, at the elevated voltage, is accumulated in the capacitor, C_2_, to power the transmitter module once this energy is higher than that needed to power the transmitter module. The transmitter module only consumes when it works every *x* cycles of time. As an application example, Enocean ECO200 is an energy harvesting module that can power the transmitter module, PTM330, in order to implement a wireless sensor. The energy output of the ECO200 (120–210 μJ) is sufficient to transmit three sub-telegrams through the PTM330 module. In this way, when the final capacitor of the converter, C_2_, is resized to 22 μF and four rectifying stages are used, our system can accumulate 121 μJ of energy, which is compatible with the operation of the transmitter, PTM330, every 22 min. 

To increase the energy generated or reduce the charging time of the capacitor, one possible solution is to connect *n* piezoelectric generators, with their respective rectifiers, in parallel. When connecting *n* devices in parallel, the final voltage at the capacitor, C_2_ (Figure 6a), will be the same, but the charge time will be *n* times shorter.

## 5. Proposed Monolithic System and Fabrication Process 

**Figure 10:**
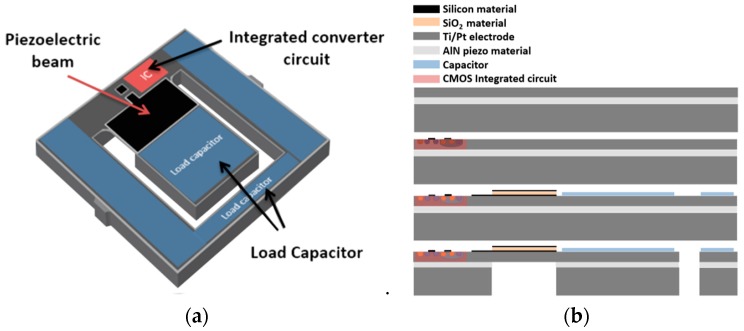
(**a)** Proposed monolithic system, composed by the converter circuit, the load capacitors, and the piezoelectric MEMS structure for energy harvesting; (**b**) cross-section sketch of the fabrication process for the proposed monolithic system.

In this work, we propose the use of a single chip composed by the energy harvester, the power management circuit, and the storage capacitors. With this monolithic integrated circuit based on a CMOS-compatible technology [33], the cost of the energy harvesting system can be reduced. In addition, taking advantage of the silicon area on top of the cantilever and the rest of the chip to add the integrated circuits and capacitors (Figure 10a), the area of the entire system can be reduced. In this way, arrays of energy harvesting systems can be incorporated in the same area, obtaining a higher power density.

Our suggestion is the fabrication of MEMS structures following a post-CMOS strategy (Figure 10b). The fabrication process starts with a CMOS standard process on an SOI wafer to create the converter circuit. The following step consists in the definition of the piezoelectric transducer and the load capacitors. The piezoelectric transducer, as already mentioned, is created when depositing, by radio-frequency (RF) sputtering, a piezoelectric layer and two metal layers that form the electrodes. Finally, a wet etch based on a buffered solution of hydrofluoric acid (BHF) is used to release the free-standing cantilever.

## 6. Conclusions

In this paper, we proposed the integration of a single chip composed of an energy harvester, power management circuit, and storage capacitors. With this monolithically integrated circuit based on a CMOS-compatible technology, the cost and the area of the entire system can be reduced.

The stress distribution in the cantilever structure of the piezoelectric devices was optimized using beams with trapezoidal shapes and rounded corners to achieve a homogeneous stress over the entire surface. With this design optimization, an increase of 60% in the power generated was obtained.

The correct operation of the charge pump was demonstrated by means of electrical simulations computed with CADENCE Virtuoso. We confirmed that a single piezoelectric generator can obtain a power density greater than 665 µW/cm^3^ for a vibration acceleration of 0.2 G at a resonance frequency of ~595 Hz. Furthermore, by connecting *n* piezoelectric devices in parallel, we obtained power outputs in the order of mW in a volume smaller than a few mm^3^.

Our integrated system with a storage capacitor, C_2_, of 22 μF allows self-powered operation in real conditions of a transmission module and its associated sensors every ~20 min.
